# Therapies from Fucoidan: New Developments

**DOI:** 10.3390/md17100571

**Published:** 2019-10-09

**Authors:** J. Helen Fitton, Damien N. Stringer, Ah Young Park, Samuel S. Karpiniec

**Affiliations:** Marinova Pty Ltd., 249 Kennedy Drive, Cambridge, Tasmania 7170, Australia; damien.stringer@marinova.com.au (D.N.S.); ahyoung.park@marinova.com.au (A.Y.P.); sam.karpiniec@marinova.com.au (S.S.K.)

**Keywords:** fucoidan, microbiome, analysis, norovirus

## Abstract

Since our last review in 2015, the study and use of fucoidan has extended in several research areas. Clinical use of fucoidan for the treatment of renal disease has become available and human safety studies have been undertaken on radiolabeled fucoidan for the purpose of imaging thrombi. Fucoidan has been incorporated into an increasing number of commercially available supplements and topical treatments. In addition, new measuring techniques are now available to assess the biologically relevant uptake of fucoidans and to assist in production. Microbiome modulation and anti-pathogenic effects are increasingly promising applications for fucoidans, due to the need for alternative approaches to antibiotic use in the food chain. This review outlines promising new developments in fucoidan research, including potential future therapeutic use.

## 1. Introduction

Fucoidans are high molecular weight, fucose-based, sulphated polysaccharides from brown macroalgae [1]. Published research regarding fucoidans has continued to develop over the last three years, and a considerable array of products containing fucoidan extracts are now available. In the last review, we described the extraction and analysis for a range of macroalgal fucoidans, in addition to uptake and biological activity [1]. This review seeks to provide updates on analysis, regulatory status and current clinical usage of fucoidans. Other recent reviews provide a focus on promising areas of bioactivity [2], anticancer activity [3] and pharmaceutical dosage forms [4]. The commercial availability of fucoidans present in cosmetics, foods and nutritional supplements has increased. A fucoidan preparation called ‘Haikun Shenxi capsules’ was approved in China in 2003, and its clinical use as a therapeutic for chronic renal failure has been described [5]. There is now regulatory approval in the US and Europe for the use of fucoidans in supplements and cosmetics. There have been new developments in assay techniques for measuring fucoidans and establishing bioavailability. The first clinical trial on intravenous delivery of a radiolabelled fucoidan has now been reported [6]. This encouraging research focusses on the use of fucoidans to image thrombi and may become the first intravenous clinical application of fucoidan. 

Despite earlier research into the potential of fucoidans to prevent post-surgical adhesions—an area of unmet clinical need—only one commercially available product for use in horses, called PERIDAN™, has eventuated [7]. A 2019 study confirmed the efficacy of both research grade *Fucus vesiculosus* and PERIDAN™ (*Laminaria japonica*) fucoidans in reducing abdominal adhesions in a rat model [8]. Interestingly, continuous infusion of either PERIDAN™ or *Fucus vesiculosus* fucoidan was sufficient to prevent adhesions, but as a single bolus, only fucoidan from *Fucus vesiculosus* was successful. At the time of writing, no human clinical trials are listed.

The effects of fucoidan on microbiome is an emerging area of focus. Global concern regarding the increase of drug-resistant superbugs and the lack of new antibiotics for treating human and animal diseases has led to a call for new approaches. In agriculture, there is an urgent need to develop strategies to replace antibiotics for food-producing animals, especially poultry and livestock. In human health, there is increasing awareness of a connection between the microbiome and disease conditions. Fucoidans have bacteria-inhibiting qualities against the ulcer-causing *Helicobacter pylori* [9,10,11], and modulate the growth and biofilm-forming properties of other types of bacteria [12]. Additional antiviral activity and the anti-inflammatory nature of fucoidans [1] make them suitable for a wide range of digestive tract applications. In particular, fucoidans can attenuate inflammation generated by lipopolysaccharides produced by Gram-negative bacteria [13]. New research demonstrates activity against norovirus, for which there are no current treatments [14]. Perhaps much of the biological activity ascribed to fucoidans may be due their effects on modulating microbiome and inflammation from the oral cavity and throughout the length of the gut. 

The use of fucoidans as potential agents in oncology has been recently reviewed by others and is briefly expanded upon here [3,15]. The mechanism by which fucoidans could induce either a direct or indirect anticancer effect is better understood. The modulation of immune activity by fucoidans shows promise, not only as an anti-inflammatory agent, but also as a potential vaccine adjuvant. This immune-modulatory effect may also represent an additional anticancer mechanism for fucoidans. Observations in elderly Japanese subjects showed that oral administration of a fucoidan extract enhanced their response to influenza vaccines [16]. The mechanism for this useful activity may be associated with the ability of fucoidans to bind to Toll-like receptors [17]. Another emerging application in the literature is the use of fucoidans in ocular diseases [18], particularly age-related macular degeneration due to their ability to interfere with the activity of vascular endothelial growth factor (VEGF) [19].

## 2. Bioavailability and Measurement of Fucoidans

Measuring fucoidans during extraction processes, and in biological tissues and fluids, remains technically challenging. Standard laboratory methods for analysis have been outlined previously [1]. Recent developments in measurements in biological fluids include an electrochemical interface method [20], and a highly sensitive dye-based method, now commercially available from the analytical company, Redprobes [21]. A summary of recent developments is outlined in Table 1.

Interestingly, some fucoidan preparations sold commercially for use in food and supplements may not contain fucoidan as stated. Some preparations appear to be alternative polysaccharides, perhaps laminarin, whilst others may be substituted with glucose or cellulose [24]. 

The oral bioavailability of fucoidan preparations is generally low, although recent studies have elegantly demonstrated the uptake and tissue distribution of *Fucus vesiculosus* fucoidan in a rat model [25]. A Japanese study has further confirmed the bioavailability of fucoidan and its excretion in urine, after ingestion of whole seaweed [26]. This follows their earlier work demonstrating the uptake of orally delivered fucoidan in serum and urine [27]. Uptake studies are outlined in Table 2. The recently described observations of clinical efficacy of orally delivered fucoidan for chronic renal failure indicate probable systemic uptake [5] in humans. Systemic uptake after oral delivery indicates potential for additional clinical applications in the future, perhaps including the control of thrombosis [28].

## 3. Regulatory Framework for the Use of Fucoidans

### Food and Supplements

In recent years, fucoidan extracts have attained regulatory approvals in a number of global jurisdictions for use in foods and dietary supplements. In particular, fucoidan extracts from *Undaria pinnatifida* and *Fucus vesiculosus* were filed as ‘Generally Recognised as Safe’ (GRAS) with the US FDA by the Australian manufacturer, Marinova. The FDA had no further questions in relation to the two GRAS determinations, which permit daily consumption of high-concentration fucoidan extracts from *Undaria pinnatifida* or *Fucus vesiculosus* at rates of up to 250 mg/day. 

In the European Union, the same fucoidan extracts from *Undaria pinnatifida* and *Fucus vesiculosus* were assessed by the European Commission and found to be substantially equivalent to the parental seaweeds from which they are extracted, and hence were approved as novel foods under the Commission Implementing Regulation (EU) 2017/2470 of 20 December 2017, for consumption up to 250 mg/day.

In Canada and Australia, the respective agencies have approved a number of listed medicines containing fucoidan extracts. In Australia, fucoidans have been approved in a species-specific context for both *Undaria pinnatifida* and *Fucus vesiculosus*. They are recognised as listable components of their parental herbal (seaweed) source.

## 4. Clinical Use of Fucoidan Extracts

The first clinical trial on intravenous delivery of a radiolabelled fucoidan has now been reported [6]. The ability of fucoidans to bind to p-selectin groups on platelets assists in the imaging of blood flow-limiting thrombi. This application may become the first non-oral clinical application of fucoidan.

Reports of orally delivered clinical use of fucoidans have increased in the last few years. A recent paper outlines the use of fucoidan from *Saccharina japonica* to address kidney disease in China [5]. This traditional Chinese medicine formulation shows promise for use in other parts of the world and is supported by studies in the literature detailing the use of fucoidan to treat kidney disease in a variety of animal models. Mesenchymal stem cells (MSCs) are a class of cells that show promise in regenerating diseased organs [30]. Currently, the damage caused to MSCs by uremic toxins makes kidney MSC transplantation unfeasible. However, fucoidan from *Fucus vesiculosus* reversed senescence caused by the uremic toxin p-cresol, indicating a potential support for both resident and transplanted MSCs [31].

Following on from an animal study showing inhibition of inflammation in ethanol-induced gastritis [32], a clinical study in young Chinese men showed a substantial—and no doubt welcome— decrease in gastritis (cause undisclosed) after ingestion of a blend of fucoidan and wheat peptides [33].

Additional studies have demonstrated fucoidans utility in reducing inflammation in cancer patients [34]. No interactions or adverse effects were observed with the commonly used hormone therapies tamoxifen and letrozole in breast cancer patients. Reductions in joint pain were also noted following co-administration of fucoidan from *Undaria pinnatifida* [35].

Fucoidan administration improved taste sensitivity in diabetics [36], whilst a study on obese, but non-diabetic, subjects using a fucoidan-polyphenol complex from *Fucus vesiculosus* showed no effects on glucose or insulin resistance [37].

Lastly, a clinical topical study using a cream containing 4% fucoidan was found to be effective for treating oral herpes [38]. This topical application is supported by a substantial body of research indicating excellent inhibitory activity against herpes viruses [39]. These recent clinical studies are further summarised in Table 3.

## 5. Biomaterials and Drug Delivery

Functionalities offered by fucoidans have been exploited in numerous experimental biomaterials. The P-selectin binding activity of a fucoidan extract has led to the development of a radiolabeled thrombus-marking preparation to enable imaging. A clinical trial was recently completed on the tolerance, biodistribution and dosimetry of fucoidan radiolabeled by Technetium-99m [6,41].

The anti-inflammatory properties of fucoidans have also been utilised in combination peptide gels [42]. The addition of a fucoidan extract aided the formation of fibrillary peptide gels which were then assessed in vitro and in vivo. These gels were used to provide a scaffold for the regeneration of brain tissue and the prevention of scar formation [43]. In similar preparations, the fucoidan extract caused apoptosis in an epithelial cancer cell line [44], indicating the potential of the gel to restrict cancer recurrence at the surgical excision site.

There is an especially rich variety of new literature in the area of drug delivery, as reviewed recently [4]. For example, fucoidan-containing films have been investigated for their potential in drug delivery systems. In one study, polyelectrolyte multilayers (PAMs) were formed using chitosan and different molecular weight fractions of *Fucus vesiculosus* fucoidan [45]. It was found that the different fucoidan fractions greatly altered the structure of the PEMs, affecting hydration, elasticity and the capacity and type of small molecules to migrate through the material. This could allow for a variety of specialised wound dressings to be developed with different affinities for proteins, or to allow for the migration of functional molecules to the wound site to aid the healing process.

Another novel drug delivery system was developed based on the self-assembly of two polyelectrolytes, poly-allylamine hydrochloride (PAH) and a fucoidan extract. This system was developed to deliver the anticancer drug methotrexate, [46]. Such drug delivery systems may eventually allow lower amounts of drug to be delivered over a sustained period of time in order to maximise anticancer effects. Enhanced immune regulation activity against breast cancer was developed using fucoidan nanoparticles containing doxorubicin [47], while another type of fucoidan-chitosan coated gold nanorod was developed for photo-initiated anticancer therapy using far infrared light [48]. These generated positive results after laser treatment of breast cancer tumours in mice. Manganese fucoidan nanoparticles reversed hypoxia-induced radiotherapy resistance by decreasing clonogenic survival and increasing DNA damage and apoptotic cell death [49]

Immobilised fucoidans on a variety of surfaces have also assisted in bone regeneration models [50] and in the prevention of coagulation [51]. A materials science application for fucoidans, in the form of immobilised fucoidan on steel surfaces, has potential to inhibit the surface adhesion and transfer of infectious agents and inhibit biofouling [52]. This novel approach may have utility in coating surgical instruments and implantable devices. Studies are summarized in Table 4.

## 6. Microbiome and Fucoidan

Oral, digestive and skin microbiomes can all be modulated by the presence of fucoidans. As research into the microbiome, virome and phageome develops, the interactions between the microbiome and disease states continue to unfold. The efficacy of drugs and checkpoint inhibitor therapy [61], responses [62], cancer [63], obesity, inflammatory disorders [64] and mental health [65] have all been associated with microbiome.

Fucoidans have been noted to create favourable changes in the microbiome [66] and additionally, to independently modulate checkpoint PD-L1 in cancer cells [67]. The microbiome and intestinal integrity in a breast cancer-bearing rat model was favourably affected by fucoidan intake [68]. These alterations could be considered as an additional potential mechanism by which fucoidans can act as a preventative to breast cancer.

Oral bacteria were affected particularly by fucoidan from *Fucus vesiculosus*, at inhibitory concentrations below 1 mg/mL. The inhibition of biofilm formation and planktonic cell growth was also observed, showing promise for the inclusion of fucoidans in dental products [12,69].

Gastric damage was markedly inhibited by a fucoidan-containing preparation in a double blind clinical study [70]. An interesting new development showed that fucoidan has the ability to prevent gastric mucosa adhesion of the highly infectious norovirus, for which there are no effective preventatives or treatments [14]. Building on earlier research demonstrating the inhibition of influenza viruses by fucoidan, a synthetic fucoidan was shown to be highly effective in binding viral hemagglutinin [71].

There is great potential for fucoidans to both modulate microbiome and prevent the binding of infectious pathogens. Studies are summarized in Table 5.

## 7. Oncology

As described previously, fucoidans appear to act both directly on cancer cells and indirectly, by increasing immune clearance of cancer cells and preventing metastasis. For example, tumour metastasis and cachexia were attenuated via the inhibition of vascular endothelial growth factor (VEGF) and matrix metalloproteases in a Lewis lung cancer tumour model mice given oral fucoidan [73].

Fucoidans act independently as checkpoint modulators and may serve as alternative complementary agents for the treatment of cancers with high PD-L1 expression [67]. Apoptosis or cell cycle arrest appears as a common feature of fucoidan bioactivity directly on cancer cells. Uterine cancer cell lines were inhibited by fucoidans derived from *Undaria pinnatifida*, whilst normal cells were unaffected [74]. Lower molecular weight fractions of *Undaria* fucoidan were highly effective inhibitors of human prostate cancer cells, when delivered orally in a xenografted mouse model [75]. Head and neck cancers, which may be caused by human papilloma viruses (HPV), are particularly difficult to treat. Fucoidan from *Fucus vesiculosus* inhibited head and neck squamous cell lines, causing cell cycle arrest [76]. It also enhanced the response to the anticancer drug cisplatin in HPV-affected lines. Preclinical evaluations of fucoidans from *Fucus vesiculosus* and *Undaria pinnatifida* in a mouse model showed activity as sole agents and additive activity with anticancer agents in some breast cancer and ovarian cancer tumours [77]. It should be noted that fucoidans do not always act as inhibitors of cancer cell lines, and were ineffective against uveal melanoma cell lines [78], instead providing protective and angiogenic effects.

The mechanisms of these anticancer activities are multifaceted. They include induction [79], or even inhibition [76], of reactive oxygen species, destabilization of mitochondria, and the cleavage of caspases and PARP. Using a systematic screen of the entire set of 4733 haploid *Saccharomyces cerevisiae* gene deletion strains, an analysis of cell pathways revealed that multiple cellular pathways were affected by exposure to fucoidan extracts, and that cDNA damage and cell cycle arrest occurred in colon cancer cell lines. Normal fibroblasts were unaffected under the same conditions [80]. There were global effects of the fucoidan studied on a wide range of eukaryotic cellular processes, including RNA metabolism, protein synthesis, sorting, targeting and transport, carbohydrate metabolism, mitochondrial maintenance, cell cycle regulation and DNA damage repair-related pathways. Thus, the mechanisms by which fucoidans may act on cancer cells is becoming clearer. The lack of effect on normal fibroblasts means that fucoidan extracts have potential utility as anticancer agents.

In a Russian study, fucoidan from *Fucus evanescens* was found to increase cancer susceptibility towards radiation [81]. The growing amount of evidence described above suggests that fucoidans place stresses on multiple cellular pathways, thereby increasing cancer cell susceptibility to radiation or other agents. Conversely, fucoidans may confer radiation protection effects that help to prevent lung damage and subsequent fibrosis of healthy tissues [82].

The immunosuppression experienced after chemotherapy can lead to infectious diseases and reduced quality of life. Previous research has shown some increase in circulating stem cells after ingestion of a fucoidan extract [83]. In a recent Russian study, semisynthetic fucoidan fractions were assessed in a cyclophosphamide immune-suppressed mouse model. A subcutaneously delivered fucoidan-derived octasaccharide was more effective in neutrophil regeneration than the gold standard peptide treatment, rG-CSF [84]. Recent research is summarized in Table 6.

## 8. Imaging and Control of Coagulation

French researchers have developed an extensive body of work regarding the use of radiolabelled fucoidan as an imaging agent to locate thrombi and image early-stage inflammatory processes in autoimmune endocarditis [86,87]. A clinical trial on the safety of the reagent was completed in 2019 [6], clearing the way for commercialization and clinical use of this imaging tool.

This work has expanded to assist in the targeting of thrombi to deliver a clinically used thrombolytic agent, recombinant tissue plasminogen activator (rtPA). A fucoidan extract is incorporated into nanoparticles which are then loaded with the rtPA. This allows the specific targeting of thrombi at lower overall doses, and may avoid some of the haemorrhagic complications of using rtPA [88]. Interestingly, fucoidans themselves are good inhibitors of the tPA-PASI1 complex, and act as thrombolytics in their own right. Combined Korean- and Russian-based research has demonstrated the utility of these fractions, building on earlier work that demonstrated anti-thrombotic activity without anticoagulant activity in a fraction from *Undaria pinnatifida*. [89]. The potential to use orally bioavailable thrombolytic approaches are attractive [28].

Dextran-coated superparamagnetic iron oxide nanoparticles or ‘SPIONs’ are in use as MRI contrast agents. The wider clinical potential of SPIONs is limited by their rapid removal from circulation via the reticuloendothelial system (RES). Fucoidan ingestion appears to increase the retention time of SPIONs by preventing their uptake into the reticuloendothelial system [90,91,92]. This useful activity expands on the applications for imaging of thrombi.

Gradually depolymerised fractions of *Fucus vesiculosus* fucoidans (without removing sulphates) were examined for bioactivity [91,92]. Researchers concluded that the fractions gradually lost their anti-oxidative and anti-proliferative activities due to the removal of terpenoids and polyphenolics. It is likely that the terpenoid and polyphenolic components were co-extracted with the initial fucoidan and subsequently removed, rather than being incorporated within the structure of the fucoidan polymer itself. Interestingly, lower molecular weight fractions maintained an anti-inflammatory activity, whilst having a low anticoagulant activity. Studies are summarized in Table 7.

## 9. Neuroprotection

One of the key issues with natural or drug agents for neuroprotection is the blood–brain barrier. Whilst systemic effects can undoubtedly translate to brain effects, any direct actions will ultimately need a delivery system into the central nervous system.

Having said that, new avenues of research into fucoidans as neuroprotective agents are promising. In a model of transient ischemia, fucoidans delivered by intraperitoneal (ip) means were found to effectively protect neurons from ischemic events through attenuation of activated glial cells and reduction of oxidative stress via increase of super oxide dismutase production [93]. In Alzheimer’s related research, a fucoidan extract inhibited the formation of amyloid fibrils in vitro, and their toxic effect on neuronal cells [94].

Various models of Parkinson’s disease treated with fucoidans have shown encouraging data [95,96]. While the mechanism behind these results is unclear, the recent findings on microbiome connections with Parkinson’s disease and many other neurological conditions point to a potential effect on gut microbiota [97]. Recent research is summarized in Table 8.

## 10. Conclusions

Fucoidans continue to be developed as bioactive oral supplements and will likely increase their market presence in the future. Gut health, oral health and anti-inflammatory applications are already partly commercialised for use in humans, livestock and pets, with the recent suite of regulatory approvals for fucoidan extracts in the US and EU likely to spur further growth in these sectors.

Promising research, even when patented, often fails to be commercialised due to lack of regulatory approval and a clear need in the market. Regulatory approval of a fucoidan preparation for chronic renal failure has provided clinically useful outcomes in China. Fucoidans have enormous potential as part of drug delivery systems and devices, and they show particular near-market potential in imaging and in treatments for thrombosis. Safety studies on radiolabelled fucoidan have been carried out with a view to regulatory approval for clinical imaging applications. Subject to regulatory approvals, fucoidans could be used in the near future.

Orally bioavailable adjunct therapies for neurological disease, bacterial and viral infections, and oncology also appear to be commercial possibilities with research now progressing into the clinical trial phase.

## Figures and Tables

**Table 1:** Recent fucoidan measurement techniques.

Fucoidan Source	Method	Outcome	Reference
*Fucus vesiculosus Undaria pinnatifida*	Electrochemical interface measuring technique	Detection at low μg mL^−1^ concentration.	[20]
Fructan fucoidan blends	FT-IRs	Food analysis and confirmation	[22]
Sea cucumber *Stichopus japonicus*	HPLC-MS/MS methods for quantitation	Able to differentiate fucosylated chondroitin sulfate and fucoidan	[23]
*Undaria pinnatifida, Macrocystis pyrifera*	Heparin red	Detection in serum	[21]

**Table 2:** Uptake and distribution of fucoidan.

Fucoidan Source	Study Type	Outcome	Reference
*Fucus vesiculosus*	Rat tissue distribution	Inverse detection method	[25]
*Fucus vesiculosus*	Mouse-fluoro labelled fucoidan	Detected by fluorescence spectrometry and HPLC	[29]
*Cladosiphon okamuranus*	Clinical study—urine and serum	Detected uptake in urine and serum	[26]

**Table 3:** Clinical studies involving fucoidan.

Fucoidan Source	Aim of Study	Type of Study	Outcome	Reference
*Saccharina japonica*	To review use of fucoidan to treat renal diseases and discuss clinical outcomes for Haikun Shenxi capsule in chronic renal failure patients	Clinical study and review	Fucoidan inhibits renal fibrosis and glomerular sclerosis by reducing the accumulation of extracellular matrix.	[5]
*Unspecified*	To establish safety of a radiolabeled fucoidan	Clinical Healthy subjects Intravenous	Fucoidan is safe. Distribution established.	[6]
*Cladosiphon sp*.	To examine the efficacy of fucoidans especially focusing on inflammation in relation to (QOL)quality of life scores for advanced cancer patients	Clinical Cancer patients	Pro-inflammatory cytokines significantly reduced after two weeks of fucoidan ingestion and QOL scores stayed.	[34]
*Cladosiphon sp.*	Absorption study	Clinical Healthy Subjects	Residents in Okinawa prefecture had significantly higher fucoidan excretion.	[40]
*Unspecified*	To evaluate the protective effect of the combination of wheat peptides and fucoidan (WPF) on adults diagnosed with chronic superficial gastritis	Clinical Chronic gastritis patients	WPF reduced gastric mucosal damage and improved symptoms and altered gut microbial profile in beneficial way.	[33]
*Cladosiphon* sp.	Physiological effects of fucoidan on glucose metabolism, the digestive system and taste sensitivity.	Clinical Patients with type-2 diabetes	Improved taste sensitivity Increased stool frequency	[36]
*Fucus vesiculosus*	To determine if fucoidan/polyphenol extract reduces insulin resistance	Clinical Overweight non-diabetic subjects	Safety affirmed. No effects on insulin, glucose.	[37]
*Undaria pinnatifida*	To investigate the effect of co-administration of fucoidan on letrozole and tamoxifen.	Clinical interaction study Breast cancer patients	Fucoidan is safe to be taken with letrozole and tamoxifen.	[35]
*Nemacystus decipiens*	To determine efficacy of 4% fucoidan cream for recurrent oral herpes labialis	Clinical Topical study Patients with cold sores	Recurrent oral herpes labialis was markedly improved by the cream in terms of both healing and time to loss of discomfort.	[38]

**Table 4:** Biomaterials and drug delivery.

Fucoidan Source	Aim of Study	Type of Study	Effect	Reference
*Unspecified*	Functionalised microbubble P-selectin markers for thrombus	In vivo	Fucoidan microbubbles were able to target thrombus specifically.	[53]
*Undaria pinnatifida*	Peptide gels for scaffolds	In vitro	Fucoidan generates fibrillary peptide gels	[42]
*Undaria pinnatifida*	Peptide gels in brain injury	In vivo	Reducing astrocytic scarring	[43]
*Undaria pinnatifida*	Peptide gel control of muscle cell morphology	In vitro	Fucoidan peptide gels reduce formation of mutinucleated syncytia in myoblasts	[54]
*Laminaria japonica*	Eggshell protein chitosan fucoidan for intestinal inflammation	In vivo	Reduced lipopolysaccharide (LPS)-induced intestinal epithelial inflammation	[55]
*Fucus vesiculosus*	Bone regenerations fucoidan peptide	In vitro	Developed a new mechanically and thermally stable bioorganic scaffold for bone tissue engineering	[50]
*Fucus vesiculosus*	Electrospun mats with fucoidan for osteoblasts	In vitro	Enhanced stability of the surface of blend nanofibers with very good cell viability	[56]
*Fucus vesiculosus*	Fish oil encapsulation	n/a	Significantly improved oxidative stability	[57]
*Fucus vesiculosus*	Targeted nanoparticles cancer therapy	In vivo	Directly induced T-cell activation and blocked the immunosuppressive PD-L1 pathways via intravenous administration.	[58]
*Laminaria japonica*	Hydrogels with fucoidan for platelet rich plasma delivery into connective tissues	In vivo	Hydrogel showed high strength, stability, strong adhesive ability and promoted cartilage regeneration in a rabbit.	[59]
*Fucus vesiculosus*	Anticoagulant plasma fucoidan on plastic surface	In vitro	Fully anticoagulant and suitable for blood contacting PET devices	[60]
*Fucus vesiculosus*	Antifouling coating of solid surfaces	In vitro	Catechol-conjugated fucoidan coating showed excellent resistance to platelets, bacteria and marine diatom adhesions.	[52]

**Table 5:** Microbiome and pathogen effects.

Fucoidan Source	Aim of Study	Type of Study	Effect	Reference
*Laminaria japonica*	Wheat peptides and fucoidan	In vivo (Rat)	Amelioration of gastric inflammation caused by ethanol	[32]
*Laminaria japonica*	Wheat peptides and fucoidan	Clinical	Reduced gastric mucosal damage in 70% subjects (*p* < 0.001). Altered microbiota composition post-intervention	[33]
*Laminaria japonica*	Microbiome in mice with DMBA-induced breast cancer	In vivo (Mice)	Increased bacteroidetes/firmicutes phylum ratio, increased tight junction proteins and lowered endotoxin	[68]
*Fucus vesiculosus* *Cladosiphon* sp.	Oral healthcare biofilms	In vitro	*Candida albicans, Streptococcus mutans*, and *Porphyromonas gingivalis*; significantly inhibited the adhesion of *S. mutans* to bovine teeth and porcelain; were suggested to bind to and neutralise endotoxin (lipopolysaccharide) in a LAL assay; and showed COX-1 and/or COX-2 inhibitory activity	[12]
*Fucus vesiculosus* *Undaria pinnatifida* *Macrocystis pyrifera* *Hizikia fusiforme Kjellmaniella crassifolia Laminaria japonica* *Sargassum hornerii*	Effects on bacterial plaque (oral cavity)	In vitro	Minimum inhibitory concentrations of 125 to 1000 μg/mL. Above 250 μg/mL completely suppressed biofilm formation and planktonic cell growths of *Streptococcus mutans* and *S. sobrinus*	[69]
Synthetic fucoidan activity	Influenza virus infection	In vitro viral MDCK plaque assay	Bound to influenza virus haemagglutinins (HAs) and inhibited haemagglutination activity.	[71]
*Kjellmaniella crassifolia*	Influenza virus infection	In vitro	Bound to and inhibited viral neuraminidase and interfered with the activation of EGFR, PKCα, NF-κB, and Akt; intranasal administration improved survival and decreased viral titres.	[72]
*Fucus vesiculosus*	Anti-norovirus	In vitro	Fucoidan prevented the binding of norovirus	[14]

**Table 6:** In vitro and in vivo cancer studies.

Source of Fucoidan	Aim of Study	Type of Study	Outcome	Reference
*Undaria pinnatifida* *Fucus vesiculosus*	Orthotopic cancers in mice	In vivo Mouse	Safety of fucoidan usage during breast cancer treatment and potential to improve tamoxifen activity.	[77,85]
Semisynthetic fucoidan fraction	Cyclophosphamide-treated mice, haemopoiesis	In vivo mouse	Synthetic octasaccharide is identified as an effective stimulator of haematopoiesis.	[84]
*Undaria pinnatifida*	Cell cycle arrest in HCT116 and MOA yeast gene deletion study	In vitro	Global effects of fucoidan on a wide range of eukaryotic cellular processes and inhibitory effect on colon cancer cells.	[80]
*Undaria pinnatifida*	Uterine carcinoma and sarcoma cell lines	In vitro	Anticancer agent activity against endometrial stromal sarcoma and carcinosarcoma.	[74]
*Fucus evanescens*	Radio sensitisation human melanoma, breast adenocarcinoma, and colorectal carcinoma cell lines	In vitro	Increased the inhibitory effect of X-ray radiation on proliferation and colony formation-activating caspases, suppressed anti-apoptotic protein and enhanced fragmentation of DNA.	[81]
*Fucus vesiculosus*	Radiation-induced lung fibrosis	In vivo mouse	Fucoidan changed the expression patterns of inflammatory cytokines and attenuated radiation-induced lung fibrosis	[82]

**Table 7:** Imaging and coagulation studies.

Fucoidan Source	Aim of Study	Type of Study	Outcome	Reference
Radiolabelled fucoidan source unspecified	Safety	Human clinical	Safe to use. Maximum activity in liver. Activity reduced to <5% after 24 h.	[6]
*Undaria pinnatifida* *Fucus evanescens* *Saccharina cichorioides* *Costaria costata Fucus vesiculosus Eisenia bicyclis*	Thrombolytic activity of fucoidan	In vivo mouse thrombosis model, iv	Fucoidans inhibit the tPA-PAI1 complex, indicating activation of plasma tissue-type plasminogen activator is a mechanism of fucoidan-mediated thrombolysis in a mouse thrombosis model	[89]
*Unspecified*	Thrombolytic therapy based on fucoidan nanoparticles with rtPA	In vivo mouse model with induced clotting, iv	Successful thrombolysis	[88]
*Laminaria japonica*	Anti-thrombotic	In vivo mouse model. Oral delivery	Lower MW fucoidan was most effective	[28]
*Fucus vesiculosus* and 18 gradually depolymerised fractions	Degraded fucoidan fractions	In vitro	Anti-inflammatory activity, however only negligible anticoagulant activity and FXII-activating potency	[91]
*Fucus vesiculosus* *Macrocystis pyrifera* *Undaria pinnatifida*	Intravenous fucoidan administration prior to SPIONs	In vivo mouse model In vitro	Increased residence time of circulating SPIONs for imaging by blocking their uptake by reticuloendothelial uptake	[90]

**Table 8:** Neuroprotection in vitro and in vivo.

Fucoidan Source	Type of Model	Outcome	Reference
*Undaria pinnatifida,* *Fucus vesiculosus*	Neuroprotection in vitro, Alzheimer’s models	Fucoidan inhibits formation of amyloid fibrils	[94]
*Fucus vesiculosus*	Neuroprotection in vivo, intraperitoneal mouse model	IP fucoidan protects against transient ischemia	[93]
*Laminaria japonica*	Rotenone Parkinson’s model in mouse	Protection of dopamine system via preserving mitochondrial function involving the PGC-1α/NRF2 pathway	[98]
Unspecified	Parkinson’s type research in vitro	Protective effects for dopaminergic neural cells	[95]
